# A mean-field approach to the dynamics of networks of complex neurons, from nonlinear Integrate-and-Fire to Hodgkin–Huxley models

**DOI:** 10.1152/jn.00399.2019

**Published:** 2019-12-18

**Authors:** M. Carlu, O. Chehab, L. Dalla Porta, D. Depannemaecker, C. Héricé, M. Jedynak, E. Köksal Ersöz, P. Muratore, S. Souihel, C. Capone, Y. Zerlaut, A. Destexhe, M. di Volo

**Affiliations:** ^1^Department of Integrative and Computational Neuroscience, Paris-Saclay Institute of Neuroscience, Centre National de la Recherche Scientifique, Gif sur Yvette, France; ^2^Ecole Normale Superieure Paris-Saclay, France; ^3^Institut d’Investigacions Biomèdiques August Pi i Sunyer, Barcelona, Spain; ^4^Strathclyde Institute of Pharmacy and Biomedical Sciences, Glasgow, Scotland, United Kingdom; ^5^Université Grenoble Alpes, Grenoble Institut des Neurosciences and Institut National de la Santé et de la Recherche Médicale (INSERM), U1216, France; ^6^INSERM, U1099, Rennes, France; ^7^MathNeuro Team, Inria Sophia Antipolis Méditerranée, Sophia Antipolis, France; ^8^Physics Department, Sapienza University, Rome, Italy; ^9^Université Côte d’Azur, Inria Sophia Antipolis Méditerranée, France; ^10^Laboratoire de Physique Théorique et Modelisation, Université de Cergy-Pontoise, Cergy-Pontoise, France

**Keywords:** asynchronous irregular, cortical dynamics, mean field, population models, spiking networks

## Abstract

We present a mean-field formalism able to predict the collective dynamics of large networks of conductance-based interacting spiking neurons. We apply this formalism to several neuronal models, from the simplest Adaptive Exponential Integrate-and-Fire model to the more complex Hodgkin–Huxley and Morris–Lecar models. We show that the resulting mean-field models are capable of predicting the correct spontaneous activity of both excitatory and inhibitory neurons in asynchronous irregular regimes, typical of cortical dynamics. Moreover, it is possible to quantitatively predict the population response to external stimuli in the form of external spike trains. This mean-field formalism therefore provides a paradigm to bridge the scale between population dynamics and the microscopic complexity of the individual cells physiology.

**NEW & NOTEWORTHY** Population models are a powerful mathematical tool to study the dynamics of neuronal networks and to simulate the brain at macroscopic scales. We present a mean-field model capable of quantitatively predicting the temporal dynamics of a network of complex spiking neuronal models, from Integrate-and-Fire to Hodgkin–Huxley, thus linking population models to neurons electrophysiology. This opens a perspective on generating biologically realistic mean-field models from electrophysiological recordings.

## INTRODUCTION

Brain dynamics can be investigated at different scales, from the microscopic cellular scale, describing the voltage dynamics of neurons and synapses (Markram et al. 2015), to the mesoscopic scale, characterizing the dynamics of whole populations of neurons (Wilson and Cowan 1972), up to the scale of the whole brain where several populations connect together (Bassett et al. 2018; Deco et al. 2015; Sanz Leon et al. 2013).

In their pioneering work (Wilson and Cowan 1972), Wilson and Cowan describe the dynamics of a population of neurons through a well-known differential equation where the input-output gain function is described by a sigmoid. This approach inspired a long-lasting research in neuroscience where population models, usually called “rate models,” permit a qualitative insight into the dynamics of a population of neurons (di Santo et al. 2018; Hopfield 1984; Sompolinskyet al. 1988; Sussillo and Abbott 2009).

Moreover, a large effort has been made to derive population descriptions from the specificity of the network model under consideration. This bottom-up approach permits to obtain a dimensionally reduced mean-field description of the network population dynamics in different regimes (Amit and Brunel 1997; Brunel and Hakim 1999; Capone et al. 2019b; di Volo et al. 2014; El Boustani and Destexhe 2009; Montbrió et al. 2015; Ohira and Cowan 1993; Renart et al. 2004; Schwalger et al. 2017; Tort-Colet et al. 2019; Tsodyks and Sejnowski 1995; van Vreeswijk and Sompolinsky 1996, 1998). On one hand, mean-field models permit a simpler, reduced picture of the dynamics of a population of neurons, thus allowing to unveil mechanisms determining specific observed phenomena (di Volo and Torcini 2018; Jercog et al. 2017; Reig and Sanchez-Vives 2007). On the other hand, they enable a direct comparison with imaging studies where the spatial resolution implies that the recorded field represents the average over a large population of neurons (i.e., a mean field) (Capone et al. 2019b; Chemla et al. 2019).

During awake states, cortical dynamics generally show asynchronous spiking activity, where individual neurons are characterized by an irregular (typically Poissonian) firing pattern (Burns and Webb 1976; Dehghani et al. 2016; Softky and Koch 1993). In this dynamical regime, so-called Asynchronous Irregular, the correlation of the network activity decays relatively quickly in time, making it possible to develop a Markovian formalism to obtain mean-field equations. The application of such a theory to binary neurons led to the derivation of dynamical equations for population rates (Ginzburg and Sompolinsky 1994; Ohira and Cowan 1993). More recently, such a theory has been extended to spiking neurons, permitting to obtain differential equations for neurons’ average activity and for higher-order moments (Buice et al. 2010; Dahmen et al. 2016; El Boustani and Destexhe 2009). In their first order, these equations are formally the same as the rate models, like the Wilson–Cowan approach, although the function linking input-output properties of populations of neurons, namely the transfer function, is more complex than a sigmoid. Indeed, in this formalism, it encompasses internal properties of the neuronal models, together with the type of synaptic interactions under consideration, to yield a population scale description. In general, such function cannot be expressed in a closed form for complex neurons, especially if some realistic ingredients like conductance-based interactions are taken into account.

In this article we present a general approach to determine the transfer function for complex models, from the Adaptive Exponential Integrate-and-Fire (AdEx) to the Hodgkin–Huxley (HH) and the Morris–Lecar (ML) models. As a result, we obtain mean-field equations for the population dynamics in Asynchronous Irregular regimes as observed in cortical regions for highly detailed models, creating a bridge between electrophysiology at the microscopic scale and the details of the famous transfer function first used by Wilson and Cowan as a sigmoid.

Finally, we test not only the ability of our mean-field models to describe spontaneous activity of the considered neuronal populations, but also their predictive power for network response to external stimuli. We show that, provided the stimuli are fairly slow, the mean-field model gives good quantitative predictions.

## MATERIALS AND METHODS

We describe here the neuronal and network models used in this study. We also introduce mean-field equations describing population dynamics and the template to estimate the transfer function that we apply to all the neuronal models under consideration.

### Network of Spiking Neurons

We consider a random directed network of *N* = 10^4^ cells, among which 80% are regular-spiking (RS) excitatory (E) and 20% are fast-spiking (FS) inhibitory (I) neurons. The connections between pairs of neurons are set randomly with a fixed probability (*P* = 0.05). Unless otherwise stated, the same network and synaptic constants are used for all the neuronal models (Hodgkin–Huxley, Adaptive Exponential Integrate-and-Fire, and Morris–Lecar). The dynamics of each node *k* follows

(1) $${\overset{˙}{\overset{¯}{x}}}_{k}=F({\overset{¯}{x}}_{k})+I_{syn},$$

where $\bar{x}$ and $F(\bar{x})$ represent the neuronal state and dynamics, the latter depending on the specific model (see the following sections). Note the notation ${\bar{x}}_{k}$, which indicates that, in general, each neuron is characterized by a vector of variables. The synaptic current impinging on the postsynaptic neurons *k*, *I_syn_*, is modeled as

(2) $$I_{syn}=(E_{e}-v_{k})G_{syn}^{e}+(E_{i}-v_{k})G_{syn}^{i},$$

(3) $$G_{syn}^{\left(e,i\right)}(t)=Q_{\left(e,i\right)}\sum {}_{n}\Theta \left[t-t_{sp}(n)\right]e^{\frac{t-t_{sp}(n)}{\tau }},$$

where *Q_e_* (*Q_i_*) is the excitatory (inhibitory) quantal conductance. The variable τ = 5 ms is the decay timescale of excitatory and inhibitory synapses and Θ is the Heaviside step function. The summation runs over the overall presynaptic spiking times *t_sp_*(*n*). For both Hodgkin–Huxley and Adaptive Exponential Integrate-and-Fire models we set *Q_e_* = 1.5 nS and *Q_i_* = 5 nS, while for the Morris–Lecar model *Q_e_* = 4 nS and *Q_i_* = 10 nS. On top of inputs coming from other neurons in the network, each excitatory and inhibitory neuron receives an external drive in the form of a Poissonian excitatory spike train at a constant firing rate *v_drive_* = 4 Hz, if not stated otherwise.

### Single Neuron Models

We describe here the neuronal models used in the rest of the paper, starting from the Integrate-and-Fire up to the Morris–Lecar and Hodgkin–Huxley models.

#### Adaptive Exponential Integrate-and-Fire model.

The dynamics of each of the AdEx neurons *i* is described by the following 2D [here ${\bar{x}}_{i}=\left(v_{i},\;w_{i}\right)$] differential equations (Brette and Gerstner 2005):

(4) $$c_{m}\frac{dv_{i}}{dt}=g_{L}(E_{L}-v_{i})+g_{L}{\Delta e}^{\frac{v_{i}-v_{t}}{\Delta }}-w_{i}+I_{syn},$$

(5) $$\frac{dw_{i}}{dt}=-\frac{w_{i}}{\tau_{w}}+b\sum {}_{t_{sp}(i)}\delta [t-t_{sp}(k)]+a(v_{i}-E_{L}),$$

where *c_m_*=150 pF is the membrane capacity, *v_i_* is the voltage of neuron *I*, and, whenever *v_i_* > *v_t_*=−50 mV at time *t_sp_* (*i*), *v_i_* is reset to the resting voltage *v_rest_* = −65 mV and fixed to this value for a refractory time *T_refr_* = 5 ms. The leak term has a fixed conductance of *g_L_* = 10 nS and the leakage reversal *E_L_* = −65 mV, if not stated otherwise. The exponential term has a different strength for regular-spiking (RS) and fast-spiking (FS) cells, i.e., Δ = 2 mV (Δ = 0.5 mV) for excitatory (inhibitory) cells. The variable *w* mimics the dynamics of spike frequency adaptation. Inhibitory neurons are modeled according to physiological insights as the FS neurons with no adaptation while the excitatory RS neurons have a lower level of excitability due to the presence of adaptation. Here we consider *b* = 60 pA, *a* = 4 nS, and τ*_w_* = 500 ms, if not stated otherwise.

#### Hodgkin–Huxley.

The dynamics of the Hodgkin–Huxley model (Hodgkin and Huxley 1952) is given by the following five-dimensional system of differential equations (Pospischil et al. 2008):

(6) $$c_{m}\frac{dv_{i}}{dt}=g_{L}\left(E_{L}-v_{i}\right)+g_{Na}m_{i}^{3}h_{i}\left(E_{Na}-v_{i}\right)+g_{K}n_{i}^{4}\left(E_{K}-v_{i}\right)+g_{M}p_{i}\left(E_{K}-v_{i}\right)+I_{syn},$$

(7) $$\frac{dn_{i}}{dt}=\alpha_{n}\left(v_{i}\right)\left(1-n_{i}\right)-\beta_{n}\left(v_{i}\right)n_{i},$$

(8) $$\frac{dm_{i}}{dt}=\alpha_{m}\left(v_{i}\right)\left(1-m_{i}\right)-\beta_{m}\left(v_{i}\right)m_{i},$$

(9) $$\frac{dh_{i}}{dt}=\alpha_{h}\left(v_{i}\right)\left(1-h_{i}\right)-\beta h\left(v_{i}\right)h_{i},$$

(10) $$\frac{dp_{i}}{dt}=\left[p_{\infty }\left(v_{i}\right)-p_{i}\right]/\tau_{p}\left(v_{i}\right),$$

with the gating functions,

(11) $$\begin{array}{l} \alpha_{n}\left(v\right)=\frac{-0.032\left(v-V_{T}-15\right)}{exp\left[-\left(v-V_{T}-15\right)/5\right]-1},\beta_{n}\left(v\right)=0.5\;exp\left[\frac{-\left(v-V_{T}-10\right)}{40}\right],\\ \alpha_{m}\left(v\right)=\frac{-0.32\left(v-V_{T}-13\right)}{exp\left[-\left(v-V_{T}-13\right)/4\right]-1},\beta_{m}\left(v\right)=\frac{0.28\left(v-V_{T}-40\right)}{exp\left[\left(v-V_{T}-40\right)/5\right]-1},\\ \alpha_{h}\left(v\right)=0.128\;exp\left[-\left(v-V_{T}-17\right)/18\right],\beta_{h}\left(v\right)=\frac{4}{1+exp\left[-\left(v-V_{T}-40\right)/5\right]}\\ p_{\infty }\left(v\right)=\frac{1}{1+exp\left[-\left(v+35\right)/10\right]},\tau_{p}\left(v\right)=\frac{\tau_{max}}{3.3\;exp\left[\left(v+35\right)/20]+exp[-\left(v+35\right)/20\right]}, \end{array}$$

where *v_i_* is the voltage and (*n_i_*, *m_i_*, *h_i_*, *p_i_*) are the corresponding gating variables of the *i*th neuron. We set the spike emission times *t_sp_* for this model to time steps in which the membrane potential *v* exceeded a voltage threshold of 10 mV. Unless stated otherwise, the membrane capacitance *c_m_* = 200 pF/cm^2^, the maximal conductance of the leak current *g_L_* = 10 mS/cm^2^, the sodium current *g_Na_* = 20 mS/cm^2^, the delayed-rectifier potassium current *g_K_* = 6 mS/cm^2^, the slow noninactivating potassium current of the excitatory (RS) neurons *g_M_* = 0.03 mS/cm^2^ and of the inhibitory (FS) neurons *g_M_* = 0 mS/cm^2^, with corresponding reversal potentials *E_L_* = −65 mV, *E_Na_* = 50 mV, *E_K_* = −90 mV, the spiking threshold *V_T_* = −53.5 mV, and τ_max_ = 0.4 s are the fixed parameter values in *Eqs. 6**–**11*.

#### Morris–Lecar.

The dynamics of the Morris–Lecar model (Morris and Lecar 1981) is described by the system of differential equations:

(12) $$c_{m}\frac{dv_{i}}{dt}=g_{L}\left(E_{L}-v_{i}\right)+g_{Ca}M_{ss}\left(v_{i}\right)\left(E_{Ca}-v_{i}\right)+g_{K}N_{i}\left(E_{K}-v_{i}\right)+I_{syn}+I_{0},$$

(13) $$\frac{dN_{i}}{dt}=\frac{N_{ss}\left(v_{i}\right)-N_{i}}{\tau_{N}\left(v_{i}\right)},$$

where *c_m_* = 2 μF/cm^2^ is the membrane capacitance, *v_i_* is the membrane potential in mV, and *N_i_* and *M_ss_* are the fraction of open potassium and calcium channels, respectively. The current *I*_0_ = 0.2 nA/cm^2^ is a reference DC external current. Spike emission times are established in the same way as for the HH model. The maximal conductances for the leakage current (L), calcium (Ca), and potassium (K) were fixed *g_L_* = 20 mS/cm^2^, *g_Ca_* = 80 mS/cm^2^, and *g_K_* = 160 mS/cm^2^, respectively. The reversal potentials are *E_L_* = −50 mV for excitatory RS neurons and *E_L_* = −70 mV for inhibitory FS neurons, *E_Ca_* = 120 mV and *E_K_* = −84 mV. The quantities *M_ss_* and *N_ss_* are modeled as

(14) $$\begin{array}{cc} M_{ss}\left(v\right) & =\;\frac{1}{2}\left[1+tanh\left(\frac{v-V_{1}}{V_{2}}\right)\right],\\ N_{ss}\left(v\right) & =\;\frac{1}{2}\left[1+tanh\left(\frac{v-V_{3}}{V_{4}}\right)\right], \end{array}$$

with

(15) $$\tau_{N}(v)=\frac{1}{2}\left[\phi cosh\left(\frac{v-V_{3}}{2V_{4}}\right)\right],$$

where *V_1_* = −1.2 mV, *V_2_* = 18 mV, *V_3_* = 2 mV, *V_4_* = 30 mV are tuning parameters that determine the half activating voltage and slope of the activation curves for calcium and potassium conductances. This choice of parameters is such that the ML neuron is set in a type II excitability class, i.e., its response to a DC current is discontinuous and the neuron firing rate increases very slowly with the injected current (data not shown).

### Mean-Field Formalism

Mean-field theory scales the analysis of interacting pointwise neurons to their macroscopic, collective, dynamics based on the moment-statistics of the system, requiring a self-averaging hypothesis for physical quantities. We make here an additional hypothesis that the biological neural network is set to asynchronous irregular dynamical regime. The latter is chosen for its biological plausibility (Destexhe et al. 2003) as observed in awake cortical states of adult mammalian brains.

We use here the master equation formalism reported by El Boustani and Destexhe (2009) providing a system of ordinary differential equations that describe the evolution of the mean and variance of the firing rate of excitatory and inhibitory neurons. The central argument for this derivation is to consider the network dynamics as Markovian on an infinitesimal (a time resolution T, typically 20 ms) scale, as in Buice et al. (2010), Ginzburg and Sompolinsky (1994), and Ohira and Cowan (1993). Moreover, such a theory is based on the assumption that neurons emit maximum one spike over the Markovian step *T*, meaning that the theory assumes relatively low firing rate of neurons, lower than 1/*T*~50 Hz (El Boustani and Destexhe 2009), as typically is the case in the asynchronous irregular regimes here investigated. The differential equations read

(16) $$T\frac{d\nu_{\mu }}{dt}=\left(F_{\mu }-\nu_{\mu }\right)+\frac{1}{2}c_{\lambda \eta }\frac{\partial^{2}F_{\mu }}{\partial \nu_{\lambda }\partial \nu_{\eta }},$$

(17) $$T\frac{dc_{\lambda \eta }}{dt}=\delta_{\lambda \eta }\frac{F_{\lambda }\left(1/T-F_{\eta }\right)}{N_{\lambda }}+\left(F_{\lambda }-\nu_{\lambda }\right)\left(F_{\eta }-\nu_{\eta }\right)+\frac{\partial F_{\lambda }}{\partial \nu_{\mu }}c_{\eta \mu }+\frac{\partial F_{\eta }}{\partial \nu_{\mu }}c_{\lambda \mu }-2c_{\lambda \eta },$$

where μ = {*e*,*i*} is the population index (excitatory or inhibitory), ν_μ_ the population firing rate, and *c*_λη_ the covariance between populations λ and η. The function *F*_μ = {_*_e_*_,_*_i_*_}_ = *F*_μ = {_*_e_*_,_*_i_*_}_(ν*_e_*, ν*_i_*) is the transfer function which describes the firing rate of population μ as a function of excitatory and inhibitory inputs (with rates ν*_e_* and ν*_i_*). At the first order, i.e., neglecting the dynamics of the covariance terms *c*_λη_, this model reduces to the well known Wilson–Cowan model, with the specificity that the functions *F* need to be obtained according to the specific single neuron model under consideration. We introduce this procedure in the next section.

### Transfer Function Estimate

The transfer function relates the firing rate of a neuron’s response to its presynaptic excitatory and inhibitory firing rates. The particular form of the transfer function is related to the dynamics describing neuronal activity. Deriving an analytical expression for the transfer function is a nontrivial endeavor due to the nonlinear character of the dynamics, e.g., through conductance based interactions. Therefore, we use here a semianalytic approach to fit a family of plausible transfer functions to the data obtained by means of numerical simulations with the desired neuron types.

This method, developed first by Zerlaut et al. (2016) on data from experimental recordings, is based on the assumption that the transfer function depends only on the statistics of the subthreshold membrane voltage dynamics, which is assumed to be normally distributed. These statistics are the average membrane voltage μ*_V_*, its standard deviation σ*_V_*, and autocorrelation time τ*_V_*. Under these assumptions the neuronal output firing rate *F_v_* is given by the following formula:

(18) $$F_{\nu }=\frac{1}{2\tau_{V}}erfc\left(\frac{V_{thre}^{eff}-\mu_{V}}{\sqrt{2}\sigma_{V}}\right),$$

where erfc is the Gauss error function and $V_{thre}^{eff}$ is an effective or phenomenological threshold accounting for nonlinearities in the single-neuron dynamics. Note that when dealing with extremely high spiking frequencies, e.g., in the case of Hodgkin–Huxley model close to depolarization block, a multiplicative factor α can be added in front of right-hand side of *Eq. 18* to permit the fitting procedure to deal with such high frequencies. In the asynchronous irregular dynamical regime, investigated in this work, neurons have relatively low firing rates (smaller than 30 Hz). Accordingly, we never use this extension (i.e., the inclusion of the factor α) apart from the *inset* of Fig. 2*B* where we fit the transfer function of the Hodgkin–Huxley model over a broad range of frequencies, including those close to depolarization block where the firing rate is around 500–600 Hz. For this case we used α = 2. In the following section we introduce how the quantities μ*_V_*, σ*_V_*, and τ*_V_* can be expressed as functions of the presynaptic excitatory and inhibitory firing rates ν*_E_* and ν*_I_*.

#### From input rates to subthreshold voltage moments.

We start by calculating the averages (μ*_Ge,Gi_*) and standard deviations (σ*_Ge,Gi_*) of the conductances given by *Eq. 3* under the assumption that the input spike trains follow the Poissonian statistics (as is indeed the case in asynchronous irregular regimes here considered). In such case we obtain (Zerlaut and Destexhe 2017a)

(19) $$\begin{array}{l} \mu_{Ge}\left(\nu_{e},\nu_{i}\right)=\nu_{e}K_{e}\tau_{e}Q_{e},\\ \sigma_{Ge}\left(\nu_{e},\nu_{i}\right)=\sqrt{\frac{\nu_{e}K_{e}\tau_{e}}{2}}Q_{e},\\ \mu_{Gi}\left(\nu_{e},\nu_{i}\right)=\nu_{i}K_{i}\tau_{i}Q_{i},\\ \sigma_{Gi}\left(\nu_{e},\nu_{i}\right)=\sqrt{\frac{\nu_{i}K_{i}\tau_{i}}{2}}Q_{i}, \end{array}$$

where *K_i,e_* is the average input connectivity received from the excitatory or inhibitory population (in our cases typically *K_e_* = 400 and *K_i_* = 100) and in our model τ*_e_* = τ*_i_* = τ (see *Eq. 3*).

The mean conductances will control the total input of the neuron μ*_G_* and therefore its effective membrane time constant $\tau_{m}^{eff}$:

(20) $$\begin{array}{l} \mu_{G}\left(\nu_{e},\nu_{i}\right)=\mu_{Ge}+\mu_{Gi}+g_{L},\\ \tau_{m}^{eff}\left(\nu_{e},\nu_{i}\right)=\frac{c_{m}}{\mu_{G}}. \end{array}$$

Here we make the assumption that the subthreshold moments (μ*_V_*, σ*_V_*, τ*_V_*) are not affected by the dynamics of the currents coming into play at the spiking time (e.g., sodium channels dynamics or the exponential term of the AdEx model). We thus consider, for all neurons, only the leakage term and the synaptic input to estimate subthreshold moments. Accordingly, we can write the equation for the mean subthreshold voltage as

(21) $$\mu_{V}\left(\nu_{e},\nu_{I}\right)=\frac{\mu_{Ge}E_{e}+\mu_{Gi}E_{i}+g_{L}E_{L}}{\mu_{G}}.$$

The final formulas for σ*_V_* and τ*_V_* follow from calculations introduced in Zerlaut et al. (2018); they read

(22) $$\begin{array}{l} \sigma_{V}\left(\nu_{e},\nu_{i}\right)=\sqrt{\underset{s}{\sum }K_{s}\nu_{s}\frac{{\left(U_{s}\cdot \tau_{s}\right)}^{2}}{2\left(\tau_{m}^{eff}+\tau_{s}\right)}},\\ \tau_{V}\left(\nu_{e},\nu_{i}\right)=\left\{\frac{\underset{s}{\sum }\left[K_{s}\nu_{s}{\left(U_{s}\cdot \tau_{s}\right)}^{2}\right]}{\underset{s}{\sum }K_{s}\nu_{s}\left[{\left(U_{s}\cdot \tau_{s}\right)}^{2}/\left(\tau_{m}^{eff}+\tau_{s}\right)\right]}\right\}, \end{array}$$

where we defined $U_{s}=\frac{Q_{s}(E_{s}-\mu_{s})}{\mu_{G}}$ and *s* = (*e*,*i*). Notice that neglecting all the currents for the generation of action potentials (e.g., sodium current) becomes a poorer assumption as the neuron activity increases. Nevertheless, we consider here Asynchronous Irregular dynamics where neurons have typically low firing rates (on the order of few Hz). Moreover, as we show in the following sections, the fitting procedure will account for discrepancies in the actual evaluation of voltage moments by permitting an accurate prediction of neuron output firing rate.

#### From subthreshold voltage moments to the output firing rate.

The quantities μ*_V_*, σ*_V_*, and τ*_V_*, obtained in the previous section, can now be plugged into *Eq. 19* when an additional relation is taken into account. This relation follows from theoretical and experimental considerations (Zerlaut et al. 2016) showing that the voltage effective threshold $V_{thre}^{eff}$ can be expressed as a function of (μ*_V_*, σ*_V_*, τ*_V_*). In Zerlaut et al. (2016), the phenomenological threshold was taken as a second order polynomial in the following form:

(23) $$V_{thre}^{eff}\left(\mu_{V},\sigma_{V},\tau_{V}^{N}\right)=P_{0}+\underset{x\in \left\{\mu_{V},\sigma_{V},\tau_{V}^{N}\right\}}{\sum }P_{x}\cdot \left(\frac{x-x^{0}}{\delta x^{0}}\right)+\underset{x,y\in {\left\{\mu_{V},\sigma_{V},\tau_{V}^{N}\right\}}^{2^{}}}{\sum }P_{xy}\cdot \left(\frac{x-x^{0}}{\delta x^{0}}\right)\left(\frac{y-y^{0}}{\delta y^{0}}\right),$$

where we introduced the quantity $\tau_{V}^{N}=\tau_{V}G_{l}/c_{m}$. We evaluated {*P*} through a fit according to simulations on single neurons activity setting first $\mu_{V}^{0}=-60$ mV, $\sigma_{V}^{0}=0.004$ mV, ${\left(\tau_{V}^{N}\right)}^{0}=0.5$, $\delta \mu_{V}^{0}=0.001$ mV, $\delta \sigma_{V}^{0}=0.006$ mV, and $\delta {\left(\tau_{V}^{N}\right)}^{0}=1$. By the fitting procedure we find the values of the P parameters for the three neuronal models considered here (additionally for each model we consider two neuronal types: RS and FS) and we report the results in Tables 1, 2, and 3. In the first part of the results section we describe the goodness of this procedure for the three considered neuronal models.

**Table 1.** Fit parameters AdEx neurons

Cell Type	$P_{0}$	$P_{\mu_{V}}$	$P_{\sigma_{V}}$	$P_{\tau_{V}}$	$P_{\mu_{V}^{2}}$	$P_{\sigma_{V}^{2}}$	$P_{\tau_{V}^{2}}$	$P_{\mu_{V}\sigma_{V}}$	$P_{\mu_{V}\tau_{V}}$	$P_{\sigma_{V}\tau_{V}}$
RS	−49.8	5.06	−23.4	2.3	−0.41	10.5	−36.6	7.4	1.2	−40.7
FS	−51.5	4.0	−8.35	0.24	−0.50	1.43	−14.7	4.5	2.8	−15.3

Values are in mV. AdEx, Adaptive Exponential Integrate-and-Fire model; FS, fast spiking; RS, regular spiking. See *Eq. 23* for parameter definitions.

**Table 2.** Fit parameters Hodgkin–Huxley neurons

Cell Type	$P_{0}$	$P_{\mu_{V}}$	$P_{\sigma_{V}}$	$P_{\tau_{V}}$	$P_{\mu_{V}^{2}}$	$P_{\sigma_{V}^{2}}$	$P_{\tau_{V}^{2}}$	$P_{\mu_{V}\sigma_{V}}$	$P_{\mu_{V}\tau_{V}}$	$P_{\sigma_{V}\tau_{V}}$
RS	−48.1	3.2	10.9	−0.32	0.98	1.1	−1.2e-3	−1.4	3.9	−0.11
FS	−51.2	1.8	−6.1	−0.86	1.6	−0.70	−11	−0.18	1.2	−1.2

Values are in mV. FS, fast spiking; RS, regular spiking.

**Table 3.** Fit parameters Morris–Lecar neurons

Cell Type	$P_{0}$	$P_{\mu_{V}}$	$P_{\sigma_{V}}$	$P_{\tau_{V}}$	$P_{\mu_{V}^{2}}$	$P_{\sigma_{V}^{2}}$	$P_{\tau_{V}^{2}}$	$P_{\mu_{V}\sigma_{V}}$	$P_{\mu_{V}\tau_{V}}$	$P_{\sigma_{V}\tau_{V}}$
RS	339	−218	−570	−1,204	41.2	970	1,724	297	186	−155
FS	−0.615	−2.56	−17.6	−164	0.83	−55	108	−7.4	24.6	288

Values are in mV. FS, fast spiking; RS, regular spiking.

## RESULTS

We present here the results of a comparison between mean-field predictions and direct simulations. We first test the technique to estimate the transfer function of single cells in AdEx, Hodgkin–Huxley, and Morris–Lecar models and then compare theoretical predictions of the mean-field to numerical simulation of sufficiently large networks of neurons.

### Transfer Function for Integrate-and-Fire Models

**Fig. 1.**
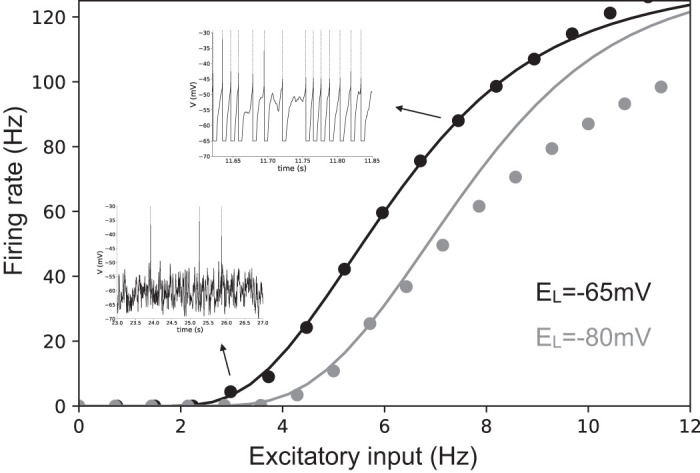
Transfer function for an Exponential Integrate-and-Fire model. Dots indicate the results of the numerical simulation of the Exponential Integrate-and-Fire model (fast-spiking cell; see materials and methods). The continuous line illustrates the results based on the semianalytic fitting. The inhibitory Poissonian spike train used here has a fixed rate of 8 Hz, while we show neuron average output firing rate as the function of the Poissonian excitatory input spike train of rate. In the *inset*s we show two exemplary voltage time traces corresponding to high (*top inset*) and low (*bottom inset*) firing rate. Colors stand for different values of the leakage reversal potential as indicated in the bottom-right corner of the figure. *E_L_*, leakage reversal.

The transfer function of a simple AdEx neuron can be straightforwardly estimated by numerical simulations. As we report in Fig. 1 its shape is very similar to a sigmoidal function (as in the seminal paper by Wilson and Cowan) but its specific parameters follow from a complex combination of microscopic information, e.g., neuron resting potential. See the black and blue dots in Fig. 1 for different values of the leakage reversal potential *E_L_*. Two main spiking modes can be distinguished in the neuronal dynamics. One is characterized by low output firing rate, where spikes are strongly driven by the membrane voltage fluctuations, namely fluctuation driven mode (see the bottom *inset* in Fig. 1). The second mode is characterized by a highly deterministic and regular firing observed at very-high-output firing rates (larger than 40–50 Hz, *top inset*). By employing the semianalytic approach to predict the transfer function we observe a very good agreement with direct simulations (see continuous lines in Fig. 1 showing predictions based on this approach) as it has been shown by El Boustani and Destexhe (2009) and Zerlaut et al. (2016). The agreement remains very good for relatively low neuronal activity (up to 50 Hz). This is a direct consequence of the semianalytic approach that assumes that neurons fire in an irregular manner (as observed in cortical dynamics) strongly driven by fluctuations around the mean membrane voltage. In this work we only consider Asynchronous Irregular population dynamics for which the activity of neurons is low, irregular and strongly fluctuation driven.

### Transfer Function for Complex Models

We report here the application of the techniques described in materials and methods to evaluating the transfer function of more complex neuronal models. To this end we consider the well-known Hodgkin–Huxley (HH) model and the Morris–Lecar (ML) models (see materials and methods). These models permit to describe the details of sodium and potassium channels dynamics neglected in the simpler Integrate-and-Fire model and reproduce time evolution of the action potential. The semianalytic approach to fit the numerical transfer function can be applied exactly in the same way as for AdEx models (as discussed in materials and methods). We consider two kinds of neurons in agreement with neurophysiological information about cortical cells: excitatory neurons modeled as RS cells with a lower gain of the transfer function and inhibitory neurons modeled as FS cells with a higher gain. A different gain of the transfer function can be obtained by changing the excitability of the cells through their resting potential or by increasing the adaptation strength (see materials and methods for details).

By comparing the theoretical prediction with numerical simulation we observe that, for the three models considered here, the transfer function is correctly estimated both for inhibitory neurons (FS cells) and excitatory neurons (RS cells). This result shows that, even by considering a much more complicated model than AdEx it is possible to have access to a semianalytic form of its transfer function and, importantly, to modify neurons excitability thus allowing to obtain a similar transfer function (of excitatory RS and inhibitory FS cells) between different models.

**Fig. 2.**
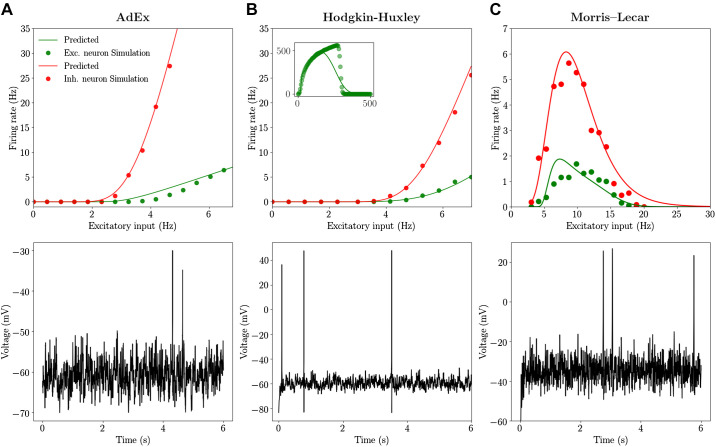
Transfer function for regular-spiking (RS) and fast-spiking (FS) cells: Adaptive Exponential Integrate-and-Fire (AdEx), Hodgkin–Huxley (HH), and Morris–Lecar (ML) models. We report the output ring rate for excitatory (Exc.) RS (green) and inhibitory (Inh.) FS (red) cells obtained from numerical simulation (dots) and from the semianalytic approach for the transfer function (continuous line). The inhibitory Poissonian spike train has a fxed rate of 8 Hz. *Bottom*: time traces of the membrane voltage of an RS cell for an excitatory input equal to 4 Hz. *Left* column is obtained for the AdEx model (*A*), *middle* column for the HH model (*B*), and *right* column for the ML model (*C*) (see materials and methods). In the *inset* of *B* we report the transfer function for the RS cell estimated over very large values of input rates. In this case a separate fit by considering a broad input frequency range has been performed (see materials and methods).

Notice that the ML model shows a decrease of firing rate at frequencies higher than 8 Hz (i.e., no voltage oscillations and thus no firing activity), as reported previously for this model by (Kim and Nykamp 2017). This is a consequence of the depolarization block (DB) observed at high input frequencies (i.e., high average external current). Accordingly, we obtain a bell-shaped transfer function, well predicted by our semianalytical formalism. In previous studies this effect was taken into account in the context of Wilson–Cowan equations by using a Gaussian transfer function, instead of a sigmoidal (Meijer et al. 2015), permitting to study the effect of the depolarization block in focal seizures at the population scale. In our model this shape, resembling a Gaussian, follows directly from Morris–Lecar equations, through the semianalytical fitting. More specifically, in Meijer et al. (2015) the DB was studied in the Hodgkin–Huxley model. Indeed, also the HH model shows a DB but, at variance with the Morris–Lecar case, it appears in our parameter setup at very high firing rates, around 600–700 Hz (see the *inset* of Fig. 2*B*). In our simulations we do not consider this dynamical regime, being far from the dynamics typical of neurons in asynchronous irregular regimes. Moreover, as described in Fig. 1, the semianalytic fitting procedure works well for low firing rates and discrepancies appear at high rates, also in the case of the simpler AdEx model. Nevertheless, we report here that, when performing the fitting over a wide range of input and output rates (see materials and methods), it is possible to obtain an overall good fit of the bell-shaped transfer function (see the *inset* of Fig. 2*B*).

Beyond the methodological point, our results show that even if the details of the mechanisms that generate a specific transfer function are very different, it is possible to adjust neuron parameters (e.g., excitability) in a way allowing to obtain similar transfer functions (at least in the region before entering a depolarization block). As a consequence, according to the mean-field theory, where what matters for the population dynamics is only the transfer function, we expect different models to have a comparable emergent dynamics at the population (collective) scale.

### Asynchronous Irregular Dynamics and Mean-Field Predictions

**Fig. 3.**
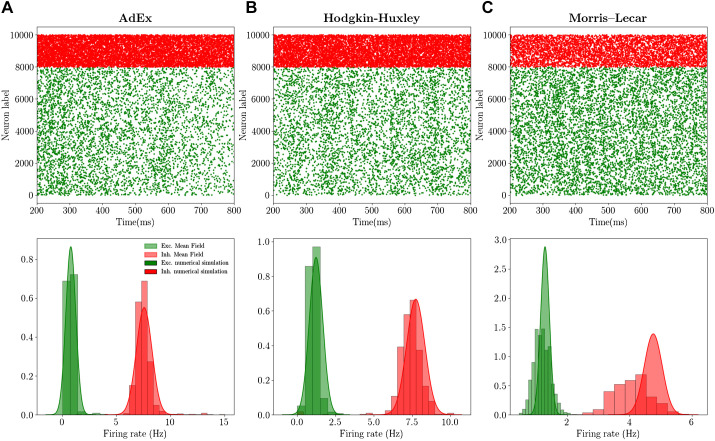
Mean-field predictions and spontaneous activity: Adaptive Exponential Integrate-and-Fire (AdEx), Hodgkin–Huxley (HH), and Morris–Lecar (ML) models. *Top*: raster plots for excitatory (green dots) and inhibitory (red dots) neurons, i.e., the spiking times for each neuron. *Bottom*: histograms (obtained on a time length Tw = 10 s) of population firing rates for excitatory (Exc.; green) and inhibitory (Inh.; red) populations. The Gaussian distribution has been plotted from mean-field predictions giving access to average firing rate and its variance. The *left* column (*A*) is obtained for the AdEx model, the *middle* column (*B*) for the HH model, and the *right* column (*C*) for the ML model (see materials and methods).

In this section we compare the mean-field predictions of the emergent dynamics of networks of AdEx, HH, and ML neurons. In particular, we simulate a sparse network of RS and FS cells (see Fig. 2) coupled through conductance based interactions (see materials and methods). By looking at Fig. 2 we observe that, before reaching the DB, all three models have similar transfer functions, with FS neurons having a higher gain with respect to RS neurons, approximately of factor 3–4. As a result, we expect the population activity in the three models to fall within a similar dynamical regime, as a natural consequence of the mean field’s sole dependence on transfer functions, previously stated. Indeed, by looking at Fig. 3 we observe that in the different networks the dynamics stabilizes on an asynchronous regime. In all cases, this regime is characterized by irregular microscopic dynamics (neuron’s spiking statistics are Poissonian, data not shown) and represents the typical spiking patterns recorded during awake states in cortical regions (the autocorrelation function of population rate decreasing to zero in the time scale of tens of milliseconds). Moreover, as expected, inhibitory FS cells fire at a higher frequency with respect to RS cells. Through the mean-field model it is possible to measure both the average population rate and its covariance (second order mean field; see materials and methods). As reported in Fig. 3 we show that the mean-field model gives a good quantitative prediction of both quantities when they are compared with the histogram obtained by sampling the population rate in the network simulation. The higher discrepancy we observe for the complex neuronal models (e.g., HH and ML case) is related to a higher mismatch of the transfer function linked to the higher complexity of the model.

### Network Response to External Stimuli

**Fig. 4.**
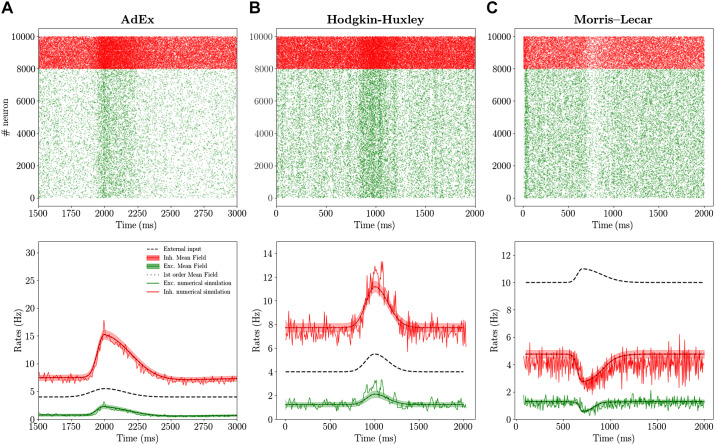
Population response to external stimuli: Adaptive Exponential Integrate-and-Fire (AdEx), Hodgkin–Huxley (HH), and Morris–Lecar (ML) models. *Top*: raster plot for excitatory (Exc.; green dots) and inhibitory (Inh.; red dots) neurons in response to an external excitatory stimulus (black dashed line in *bottom* panels). *Bottom*: corresponding population rate (noisy line) together with the mean and standard deviation over time predicted by the the second order mean-field model (red for inhibition and green for excitation). Superimposed the result obtained for the mean field at the first order (black dots), which are almost coincident with results at the second order. *Left* column is obtained for the AdEx model (*A*), *middle* column for the HH model (*B*), and *right* column for the ML model (*C*). Parameters are the same as in Fig. 3 and the external input (see *Eq. 24*) has parameters A = 2 Hz, T1 = 100 ms, T2 = 150 ms for AdEx and HH and A = 2 Hz, T1 = 100 ms, T2 = 150 ms for ML, with *t*0 = 2 s (see *Eq. 24* for parameter definitions).

To complete the comparison between the mean-field model and the network dynamics, we study the response of the system to external stimuli. In particular, we consider an incoming Poissonian train of spikes characterized by time-varying frequency and targeting both excitatory and inhibitory cells according to the following equation:

(24) $$\nu \left(t\right)=A\left(\Theta \left(t_{0}-t\right)e^{\frac{-{\left(t-t_{0}\right)}^{2}}{T_{1}^{2}}}+\Theta \left(t-t_{0}\right)e^{\frac{-{\left(t-t_{0}\right)}^{2}}{T_{2}^{2}}}\right),$$

where Θ is the Heaviside function and *T*_1_ and *T*_2_ are the rise and decay time constants, respectively. In Fig. 4 we report the comparison between the mean-field prediction and the network dynamics. By looking first at the AdEx and HH models, we observe that both mean-field models under investigation compare favorably with their corresponding network dynamics. We also verified, as it has been shown in di Volo et al. (2019), that the faster the input dynamics is, the worse the agreement becomes. Indeed, for the Markovian hypothesis to hold, we need the time scale *T* to be much larger than the autocorrelation time in the spontaneous activity T ~ τ*_m_* ~ 10 ms.

Considering now the case of the ML model, we observe by looking at Fig. 2 that a relatively strong input would bring single neurons to a depolarization block, which appears at relatively low activity levels. According to this difference with respect to AdEx and HH models, we would expect the population dynamics to show different properties in response to external perturbations. Indeed, as reported in Fig. 4*C*, the response to an external stimulus is very different from the one observed in the HH and AdEx models. In fact, in this case the excitatory stimuli turns out to inhibit both population activities. This anticorrelation between population input and output is well captured in its time course also by the mean-field model. This result shows that also for a more complex and highly nonlinear setup the mean-field model is capable of predicting the ongoing activity and the time course of the response of a network of neurons operating in the asynchronous irregular dynamical regime.

Finally, we compare the results of the first and second order mean field on average population rates. In Fig. 4 we superimpose the continuous green (red) line for excitatory (inhibitory) rate obtained with the second order mean file with the results obtained with the first order (black dots). We observe that the two quantities almost overlap (the difference is too small to be appreciated at this scale). Nevertheless, it is worth noticing that the second order mean field permits to obtain nontrivial information on the population dynamics and its fluctuations in time, with good quantitative predictions of the covariance of population rates (see the histograms in Fig. 3 and shadows in Fig. 4).

## DISCUSSION

In this paper, we have reviewed a formalism to derive mean-field models from networks of spiking neurons and we have applied it to different complex neuronal models. The key to derive such “biologically realistic” mean-field models is to be able to obtain the transfer function of complex neuronal models. The approach we followed used a mean-field formalism based on a master equation, which is applicable to every neuron, provided the transfer function is known (El Boustani and Destexhe 2009). More recently, we have shown that the usual mathematical form of the transfer function, known analytically for the Integrate-and-Fire model, can capture more complex neuronal models (Zerlaut et al. 2016, 2018). This gave rise to a “semianalytic” approach, where the transfer function is parameterized and fit numerically to the neuron model, while the mean field remains analytic as only the parameters are obtained from the fitting. This approach was applied to the AdEx model (di Volo et al. 2019; Zerlaut et al. 2018), and we extend it here to more complex models, namely the Morris–Lecar and the Hodgkin–Huxley models.

It is important to note that we are limited here to “simple” firing patterns, i.e., neurons fire tonically in response to an external stimulus. In this setup the transfer function is well defined as the neuron’s firing rate defines completely the spiking pattern. In cases where neurons exhibit different kind of activity, e.g., bursting, a different approach needs to be employed (see Ostojic and Brunel 2011). Nevertheless, in the context of tonic neuronal activity the method is shown to be able to capture the response function of highly realistic models. We have studied here the predictions of the considered mean-field models on networks dynamics of excitatory RS and inhibitory FS cell populations during asynchronous irregular regimes, as observed in awake cortical activity. The results positively compare in the case of Morris–Lecar and Hodgkin–Huxley models for both the average and the variance of network population activity.

The good predictions at the population levels in the framework of the asynchronous irregular regimes are strongly dependent on the goodness of the fitting procedure for single neurons transfer functions. Even if such procedure works very well for neurons working in a low rate regime, whenever the firing rate becomes very high (higher than 100 Hz) the quantitative agreement gets worse. A more refined technique for the evaluation of the transfer function in different states (low and high rates activity) is an important topic for future research [recent work has addressed this issue in AdEx model (Capone et al. 2019a)]. A step forward in this direction can be important when dealing with neurons entering depolarization block at high firing rates, a mechanism playing an important role in focal seizures (Meijer et al. 2015) or in dopaminergic neurons under normal condition or under drugs assumption (di Volo et al. 2019; Dovzhenok and Kuznetsov 2012). In both the Morris–Lecar and Hodgkin–Huxley models, the semianalytic fitting is found to give quite good predictions on the presence of depolarization block, especially in the Morris–Lecar case as this setup does not consider very high spiking frequencies. Even if work remains to be done to extend this framework to obtain a more reliable quantitative prediction on the depolarization block at very high frequencies, these preliminary results indicate the possibility to use these mean-field techniques to connect the physiology at the cellular scale with pathological states at the population level, as the case of focal seizures.

We also reported that, in the framework of the Asynchronous regimes here considered, corrections to first order mean field due to second order terms (see *Eqs. 16* and *17*) were relatively small but gave a good quantitative indication on the covariance of population rates (see histograms in Fig. 2). Nevertheless, in the case of dynamical regimes with higher neuronal correlation with respect to the ones here considered, we expect the second order mean field (explicitly taking into account the dynamics of covariances) to play an important role in the prediction of population average collective dynamics. The goodness of the mean-field prediction depends indeed also on the emergent dynamics of the network, i.e., in a highly synchronous dynamical regime the Markovian hypothesis fails and the mean-field model cannot give accurate predictions. Nevertheless, even if light synchronization is considered, e.g., during slow-wave sleep, the mean-field models have been shown to correctly predict such collective oscillations (di Volo et al. 2019). In this case it is, however, necessary to consider a mean-field model that includes the slow dynamics of spike frequency adaptation or that of the *I_M_* current in the case of Hodgkin–Huxley model. The possibility to include a conductance based adaptation to this formalism, e.g., by considering the slow dynamics of *I_M_* current, is a stimulating perspective for future works and will permit to obtain mean-field models for realistic neuronal models beyond the asynchronous irregular regime.

Moreover, beyond the input-output transfer function used here, a more complex transfer function has been used to take into account other features of neuron response, e.g., response in frequency (Ostojic and Brunel 2011). The addition of variables to account for a richer spiking pattern is an interesting direction, in case one is interested in modeling brain regions characterized by nontonic firing of neurons (e.g., bursting cells in the thalamus). The general framework presented here could be extended in this direction, as it has been done to account for spike frequency adaptation yielding slow oscillations at the population scale.

Another possible extension is to apply the same formalism to complex neuronal models that include dendrites. A first attempt has been made in this direction (Zerlaut and Destexhe 2017b) by considering simple “ball and stick” neuron models, where some analytic approximation is possible. In principle, it should be possible to apply this approach to models based on morphologically reconstructed neurons and to calculate the transfer function of such models. This will lead to mean-field models based on morphologically realistic neuronal models. However, the presence of dendritic voltage-dependent currents complicates this approach and should be integrated in the formalism. This suggests an exciting future development of our approach.

Finally, the positive results obtained here for complex models, by showing the generality of our approach, motivate the future step of the application of this technique directly to experimental data. To this end, neurons must be recorded intracellularly in the absence of network activity (as typically in vitro), and many combinations of excitatory and inhibitory inputs must be injected as conductances (using the dynamic-clamp technique). The first attempt of this sort was realized on the layer 5 neurons from mouse primary visual cortex (Zerlaut et al. 2016), where the transfer function could be reconstructed for a few dozen neurons. The same dynamic-clamp experiments should be done in the future to characterize the transfer function of inhibitory interneurons. Based on such experiments, it will be possible to obtain a mean-field model based on the properties of real neurons. Such a model will evidently be more realistic than the models we have presented here, which must be considered as a first step toward a quantitative population modeling of cerebral cortex and other brain regions.

## GRANTS

Research supported by the Centre National de la Recherche Scientifique and the European Union (Human Brain Project H2020-720270 and H2020-785907). M. Jedynak acknowledges support from the European Research Council under the European Union’s Seventh Framework Programme (FP/2007-2013)/ERC Grant Agreement No. 616268 F-TRACT and the European Union’s Horizon 2020 Framework Programme for Research and Innovation under Specific Grant Agreement No. 785907 (Human Brain Project SGA2).

## DISCLOSURES

No conflicts of interest, financial or otherwise, are declared by the authors.

## AUTHOR CONTRIBUTIONS

A.D. and M.d.V. conceived and designed research; M.C., O.C., L.D.P., D.D., C.H., M.J., E.K.E., P.M., S.S., and M.d.V. performed experiments; M.d.V. analyzed data; Y.Z., A.D., and M.d.V. interpreted the results of experiments; M.C., O.C., L.D.P., D.D., C.H., E.K.E., P.M., S.S., and M.d.V. prepared figures; M.d.V. drafted manuscript; M.C., O.C., L.D.P., D.D., C.H., M.J., E.K.E., P.M., S.S., C.C., Y.Z., A.D., and M.d.V. edited and revised manuscript; M.C., O.C., L.D.P., D.D., C.H., M.J., E.K.E., P.M., S.S., C.C., Y.Z., A.D., and M.d.V. approved final version of manuscript.
